# The effect of low level leukocytospermia on oxidative stress markers in infertile men

**DOI:** 10.1186/1471-2164-15-S2-P56

**Published:** 2014-04-02

**Authors:** Saad Alshahrani, Ashok Agarwal, Mourad Assidi, Adel M Abuzenadah, Ahmet Ayaz, Rakesh Sharma

**Affiliations:** 1 Center for Reproductive Medicine, Cleveland Clinic, Cleveland, Ohio 44195, USA; 2 Salman Bin Abdulaziz University, College of Medicine, Saudi Arabia; 3 Center of Excellence in Genomic Medicine Research, KAU, Saudi Arabia; 4 KACST Technology Innovation Center for Personalized Medicine, King Abdulaziz University, Jeddah, Saudi Arabia

## Background

Leukocytospermia is defined as presence of ≥1 x 106 WBC/mL of the seminal ejaculate. The World Health Organization (WHO) recommends peroxidase staining as the standard method for the detection of semen leukocytes [1,2]. The incidence of leukocytospermia ranges from 10 - 20% among infertile men. Both morphologically abnormal spermatozoa and leukocytes produce reactive oxygen species (ROS). The polymorphonuclear neutrophils and macrophages are the main components of seminal leukocytes which can generate significantly higher (>100-fold) quantities of ROS, overwhelming the ROS-scavenging mechanisms in seminal plasma and resulting in oxidative stress and damage to spermatozoa. The presence of very few activated leukocytes can produce a detectable amount of ROS [3,4]. Therefore, even a very low number of leukocytes in the sperm suspension may influence the integrity of sperm and, consequently, the outcome of assisted reproduction treatment [5]. Leukocytes contributed directly to ROS production and release and indirectly through the leukocyte-stimulated sperm. Such stimulation may be via direct contact or mediated by soluble products released by the leukocytes. The goal of our study was to assess the effect of low level leukocytospermia on semen quality and oxidative stress markers in infertile men.

## Materials and methods

In this prospective study, 211 infertile patients with no history of genital tract infections or varicocele were included. Semen samples were examined for sperm concentration, motility, seminal leukocyte levels (Endtz test) [2], reactive oxygen species (ROS) by chemiluminescence assay, and sperm DNA damage by TUNEL test. Patients were divided into 3 groups based on their seminal leukocyte levels. Group 1: no seminal leukocytes (n = 153); group 2: low level leukocytospermia (0.1 -1 X 106 WBC/mL; n = 22); and group 3: leukocytospermia (>1 X 106 WBC/mL; n = 36).

## Results

22 patients (10%) had high and 36 (18.3%) had low seminal leukocytes levels (Table 1). Conventional semen parameters between the 3 groups were similar. Patients with low level leukocytospermia had significantly higher levels of ROS (P = 0.001) and sperm DNA damage (P <0.05) compared to non leukocytospermic group. There was no significant difference in ROS levels between the two groups of leukocytospermia (groups 2 and 3).

**Table 1:** Semen parameters and its association with leukocytospermia

Parameters	Seminal leukocytes (X 10^6^ WBC/mL)		
	
	No leukocytes	0.1 – 1 WBC	> 1 WBC
Concentration (X 10^6^/mL)	53.04 ± 56.76	69.04 ± 80.72	39.35 ± 39.98

Motility (%)	48.37 ± 17.42	47.33 ± 25.74	49.23 ± 19.56

Normal morphology (%)	3.42 ± 3.12	3.56 ± 3.16	4.14 ± 3.79

ROS (RLU/ sec)	116.7 (49; 550.3)	944.8 (127; 3315.4)^a^	61286.8 (6905; 234876)^a,b^

DNA damage (%)	19.89 ± 17.31	26.47 ± 19.64^a^	24.60 ± 17.47

Results are presented as mean ± SD for all the parameters except ROS which is presented as median (25^th^; 75^th^ percentile).

^a^*P* <0.05 statistically significant compared to non leukocytospermic group.

^b^*P* <0.05 statistically significant compared to low level leukocytospermic group.

## Conclusions

Patients presenting with low levels of leukocytes have a high oxidative stress. Although these patients are not categorised as leukocytospermic by current WHO guidelines, however these men may benefit by treatment with antibiotics or antioxidant supplements to reduce ROS induced sperm DNA damage and improve therefore their chances of fertility.
